# Deep Learning Based Staging of Bone Lesions From Computed Tomography Scans

**DOI:** 10.1109/access.2021.3074051

**Published:** 2021-04-20

**Authors:** SAMIRA MASOUDI, SHERIF MEHRALIVAND, STEPHANIE A. HARMON, NATHAN LAY, LIZA LINDENBERG, ESTHER MENA, PETER A. PINTO, DEBORAH E. CITRIN, JAMES L. GULLEY, BRADFORD J. WOOD, WILLIAM L. DAHUT, RAVI A. MADAN, ULAS BAGCI, PETER L. CHOYKE, BARIS TURKBEY

**Affiliations:** 1 Molecular Imaging Branch, National Cancer Institute, National Institutes of Health, Bethesda, MD 20892, USA; 2 Department of Radiology, Northwestern University, Chicago, IL 60611, USA

**Keywords:** Bone lesion, classification, CT scan, deep learning, lesion-aware data stratification

## Abstract

In this study, we formulated an efficient deep learning-based classification strategy for characterizing metastatic bone lesions using computed tomography scans (CTs) of prostate cancer patients. For this purpose, 2,880 annotated bone lesions from CT scans of 114 patients diagnosed with prostate cancer were used for training, validation, and final evaluation. These annotations were in the form of lesion full segmentation, lesion type and labels of either benign or malignant. In this work, we present our approach in developing the state-of-the-art model to classify bone lesions as benign or malignant, where (1) we introduce a valuable dataset to address a clinically important problem, (2) we increase the reliability of our model by patient-level stratification of our dataset following lesion-aware distribution at each of the training, validation, and test splits, (3) we explore the impact of lesion texture, morphology, size, location, and volumetric information on the classification performance, (4) we investigate the functionality of lesion classification using different algorithms including lesion-based average 2D ResNet-50, lesion-based average 2D ResNeXt-50, 3D ResNet-18, 3D ResNet-50, as well as the ensemble of 2D ResNet-50 and 3D ResNet-18. For this purpose, we employed a train/validation/test split equal to 75%/12%/13% with several data augmentation methods applied to the training dataset to avoid overfitting and to increase reliability. We achieved an accuracy of 92.2% for correct classification of benign vs. malignant bone lesions in the test set using an ensemble of lesion-based average 2D ResNet-50 and 3D ResNet-18 with texture, volumetric information, and morphology having the greatest discriminative power respectively. To the best of our knowledge, this is the highest ever achieved lesion-level accuracy having a very comprehensive data set for such a clinically important problem. This level of classification performance in the early stages of metastasis development bodes well for clinical translation of this strategy.

## I. INTRODUCTION

Prostate cancer is the most common non-cutaneous malignancy in men and the second leading cause of cancer death in the United States [1]. Surgery or radiation as curative treatments can be effective only if applied when the disease is still localized to the prostate [1], [2]. Metastases outside the prostate can occur at diagnosis or as a result of tumor recurrence after therapy. Under such circumstances, the cancer cells become highly invasive, spreading from prostate to other parts of the body [1]. The lymphatic system, lung, liver, and most importantly bone are the major targets of metastatic cancer cells [3], [4]. In the prostate cancer patient population, distant metastases most commonly occur in the bones, as they provide a favorable environment for localization and formation of the metastatic tumors. Advanced prostate cancer is characterized by skeletal metastases where metastatic cancer cells enter the marrow within the center of the bone to start a vicious cycle of bone destruction and resorption.

### A. CLINICAL PROBLEM

**FIGURE 1.**
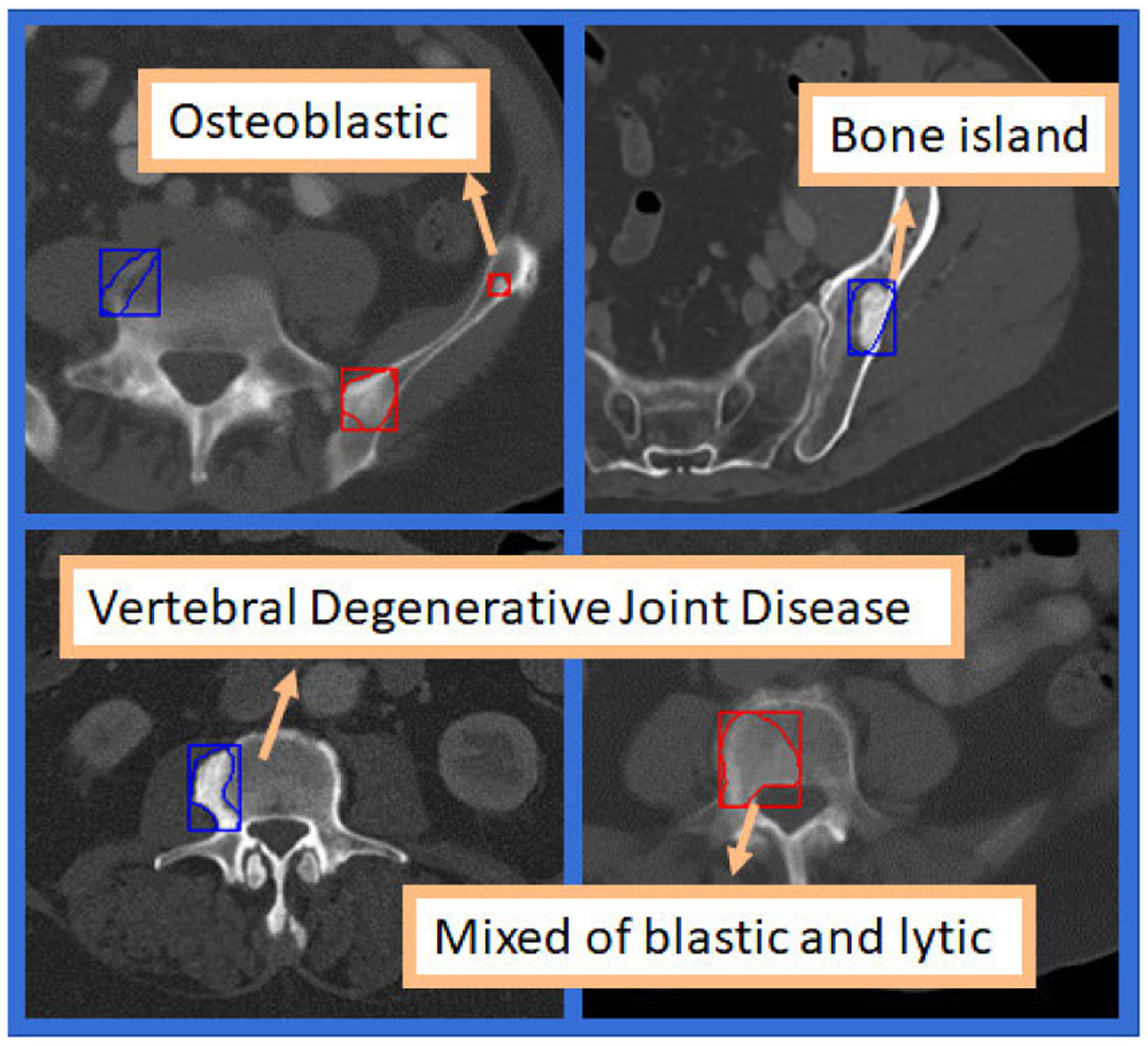
Lesions in their boundary-touching rectangular boxes demonstrated in blue and red color to signify their label as benign and malignant respectively.

Bone lesions are categorized into two major groups of benign or metastatic. There are a significant number of benign lesion sub-categories, that may overlap in appearance, including but not limited to degenerative joint disease, Schmorl’s nodules, bone islands, exostosis, deformities, old fractures, fibrous dysplasia, or hemangiomas (Figure1). On the other hand, metastatic bone lesions are classified into three sub-categories: 1) Osteolytic, 2) Osteoblastic, and 3) mixed (both osteolytic and osteoblastic). Each category can be recognized by their radiographic features [1]. To better characterize each group, one must know that bone cells exist in two different kinds, osteoblast and osteoclast, which are specialized cells involved in new bone formation and bone dissolution, respectively. Osteolytic lesions are formed through a causal chain of biochemical interactions that trigger bone resorption and promote the release of growth factors by bone matrix [1]. Such growth factors facilitate survival of metastatic bone tumor cells and the over-production of bone resorption factors. Thereby, a malicious loop of bone destruction and resorption occurs, manifested by translucent, cortical destruction on CT [1], [3]. In contrast to osteolytic lesions, osteoblastic lesions may appear hyperdense due to increased activity of osteoblast cells that form new bone tissue of poor quality around the metastatic deposit. Mixed bone metastases contain elements of osteoblastic and osteolytic features. All three types of bone metastasis increase local bone destruction, compromising the skeletal structure with frequent bone-related incidents, that can negatively impact a patient’s quality of life. Most patients with advanced prostate cancer will experience complications from bone metastases (with prevalence of up to 70%) including pain and fractures that can be debilitating [1], [3]. Metastasis to bone which commonly happen in form of osteoblastic lesions is a major cause of morbidity in these patients.

### B. IMAGING-BASED STAGING

Accurate staging of the extent of disease is a critical step in determining the appropriate therapeutic strategy for patients with prostate cancer. Patients with suspected metastases or those with suspected aggressive disease at high risk of metastasis undergo staging evaluation in form of comprehensive imaging which helps to detect or exclude bone metastases [4]. Conventional staging CT, which was originally utilized for monitoring the bone lesions, is used to evaluate lymph node or visceral spread in addition to bone metastases and it is still considered as a useful imaging technique for this purpose [3], [4]. CT enables a detailed anatomic evaluation of the skeleton, suitable for the detection of metastases. While the advent of hybrid techniques, such as PET/CT with new tracers (e.g. prostate specific antigen [PSMA] targeting tracers) contribute a component of functional imaging, such options are investigational, expensive, and have limited availability worldwide. Alternatively, conventional CT scans are quick, easy to perform, relatively low cost and widely accessible with acceptable comparative performance to demonstrate bone metastases. Consequently, in most practical scenarios of patient evaluation, CT is the modality of choice for cancer staging and for serial follow-up imaging. Recognizing malignant bone lesions from normal bone in CT scan is a challenging task, given that there are a significant number of benign bone lesion sub-categories (Figure1). It is crucial to avoid misclassification of a bone lesion as benign or metastatic since either could lead to therapeutic miss-management. Therefore, a highly accurate tool is desired to enhance the accuracy of bone staging as the results significantly impact the patient’s quality of life, course of disease, decisions about treatment, and prognosis.

### C. SNAPSHOT OF WHAT WE PROPOSE

We present a new automatic image analysis strategy for characterizing the extracted bone lesions as benign or malignant. We experimentally demonstrated the benefits of utilizing an artificial intelligence (AI) based clinical decision support system to recognize malignant lesions, given the suspicious areas from CT scans by either a radiologist or a detection algorithm. Having such an algorithm, we aim for early recognition of metastatic lesions, detailed identification of their extent, and potential improvement for on-time treatment. In this work:

- We introduced a valuable dataset to address the clinically important problem of staging the bone metastases in prostate cancer patients.
- We investigated how lesion-aware data stratification, can increase the generalizability of our binary lesion classifier model.
- We explored the impact of lesion texture, morphology, size, location, and volumetric information to better understand the learning process of an AI-based classifier in this context.
- We compared the quality of binary lesion classification using the state-of-the art classification algorithms from ResNet family [5], [6] and proposed an ensemble of lesion-based average 2D ResNet-50 and 3D ResNet-18 to address the problem of bone lesion classification.

### D. STATE OF THE ART

Cancer patient staging for early diagnosis of bone metastasis is a frequently visited topic in medical image analysis. Various clinical research focused on the same task defined at different levels using three different imaging modalities. First group of studies based their analysis on the whole-body bone scans; Sadik *et al.* utilized artificial neural network to determine the presence or absence of metastases at both lesion- and patient-level [7]. Using hand crafted feature vectors to describe the hot spots on the whole-body bone scans of both breast and prostate cancer, they achieved 90% sensitivity and 89% specificity at patient-level [7]. Dang *et al.* employed an ensemble patch-based CNN model to detect metastatic hotspots on bone scans with an accuracy of 99% [8]. In another study, Aslantas *et al.* used image gridding for feature extraction from bone scans followed by an artificial neural network classifier to determine metastases at lesion-level who achieved the accuracy, sensitivity, and specificity of 92%, 94%, and 87%, respectively [9]. Papandrianos *et al.* proposed a CNN-based architecture for diagnosis of prostate cancer metastasis from bone scintigraphy images and achieved an accuracy and average sensitivity of 97% and 95% both at the patient-level [10]. The second group of studies exploited PET-CT images to draw a conclusion regarding patients’ metastatic status. In this regard, Bradshaw *et al.* utilized VGG-19 to categorize look-alike uptakes in NaF PET/CT images into 5 classes, 1 being definitely benign to 5 being definitely malignant. They achieved accuracy and F1-score of 88%, and 87%, using lesion patches with 3-channels: maximum intensity projection (MIP) of the coronal PET, MIP of the axial PET, and axial CT [11]. In another work, Kawauch *et al.* used FDG PET-CT images to train a CNN classifier which determines whether patients need further physician’s diagnosis or not with an accuracy of 93.2 ± 3.9% for correct classification [12]. Similarly, Furuya *et al.* developed ResNet24 configuration to classify whole-body FDG PET images into 3 groups of benign, 2) malignant, 3) equivocal with an average accuracy of 97% [13], [14]. These researchers further confirmed the performance of their classification algorithm using Grad-cam [14], [15]. Recently, Kawuachi *et al.* developed a ResNet-based architecture that can classify whole-body FDG PET as 1) benign, 2) malignant or 3) equivocal with an average accuracy of 95%. The third group of studies used CT images for bone lesion categorization [16]–[18]. Similar to [19], Chmelik *et al.* used a stack of corresponding axial, coronal, and sagittal slices from the lesion volume to train a 2-D CNN that classifies vertebra metastatic lesions into lytic and sclerotic with true positive rate of 80% and 92% and False positive rate of 31, 45 respectively [20]. There is also a great body of literature that highlights image classification utilizing CT images for other organs such as lung nodule categorization, pulmonary nodule classification, and liver lesion staging [21]–[25].

## II. METHODS

**FIGURE 2.**
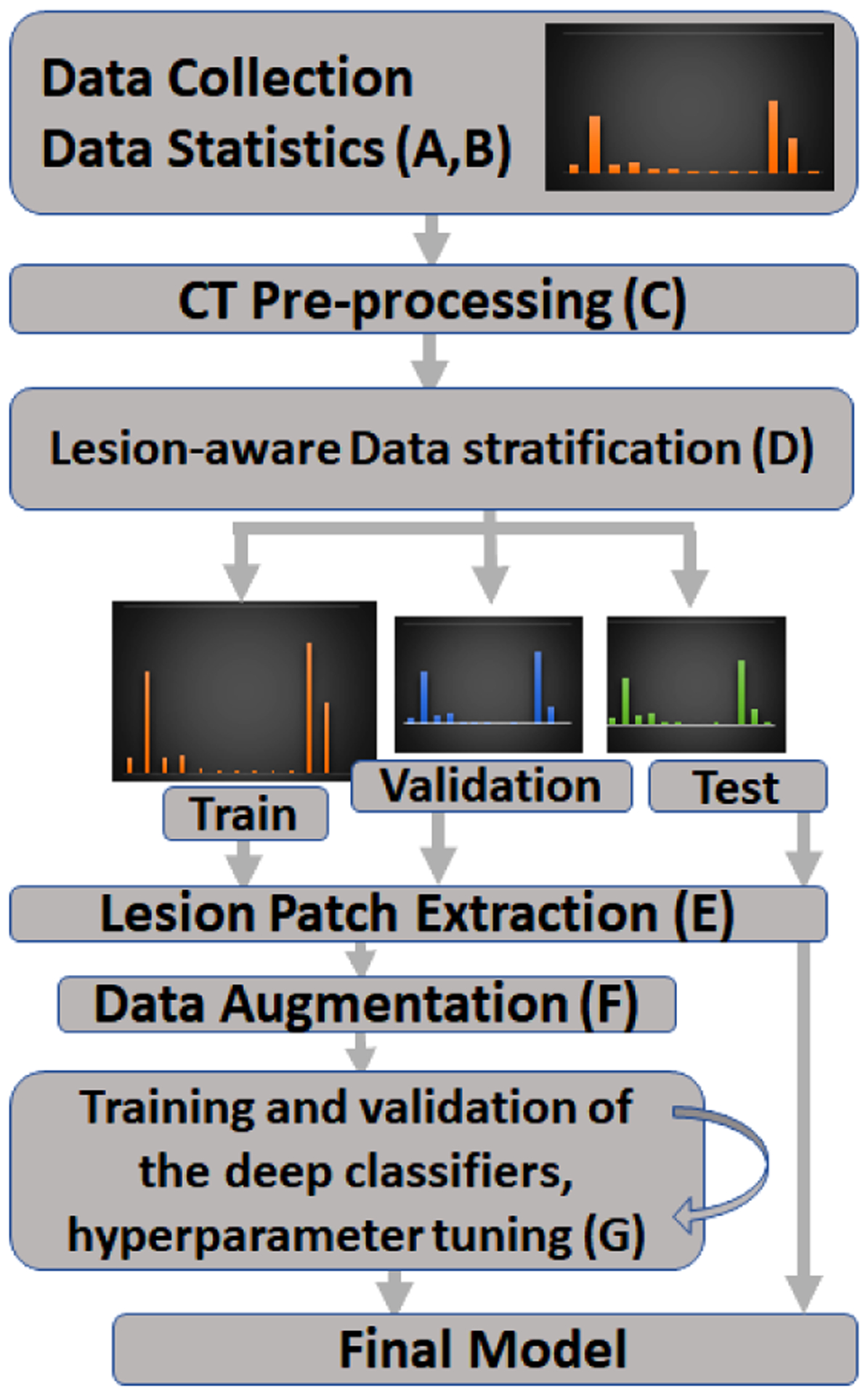
General flowchart.

In this work we are focused on benign vs malignant classification of lesions extracted from the CT images of prostate cancer patients using deep neural networks. The following sections describe our approach in developing such a model summarized by Figure 2.

### A. DATASET

Our dataset includes the bone lesion annotations obtained from staging CT scans of 114 prostate cancer patients with 41 of them histopathologically confirmed to be metastatic. An expert radiologist (17 years in body imaging) extracted these lesions in the form of full 3D segmentations (Figure 1) and labeled them as either benign or malignant. In addition to these two major categories, lesions collected in this dataset are assigned to a wide variety of lesion sub-categories. Benign lesions belong to a wide spectrum of pathologies such as degenerative joint disease, Schmorl’s nodules, bone islands, exostosis, deformities, fractures, fibrous dysplasia, hemangiomas and others (lipoma, avascular necrosis, enchondroma, etc.). Similarly, malignant lesions include osteoblastic, mixed osteoblastic-osteolytic, and osteolytic lesions. Note that throughout this paper, “*lesion*” refers to the volumetric connected area of voxels incorporating an abnormality as opposed to “*patches*” or “*lesion patches*” which are used to describe a single slice (2D) or 3 subsequent slices from a lesion (2.5D). The average bounding volume of bone lesions in our dataset is 28.52mm × 29.53mm × 13.1mm. With a z-spacing equal to 1mm, we extracted 37,685 lesion patches for 2D and 2.5D analysis. We believe that this is the first dataset of bone lesions in the field that includes full 3D segmentations and detailed labels containing a wide variety of lesion sub-categories.

### B. DATA STATISTICS

**FIGURE 3.**
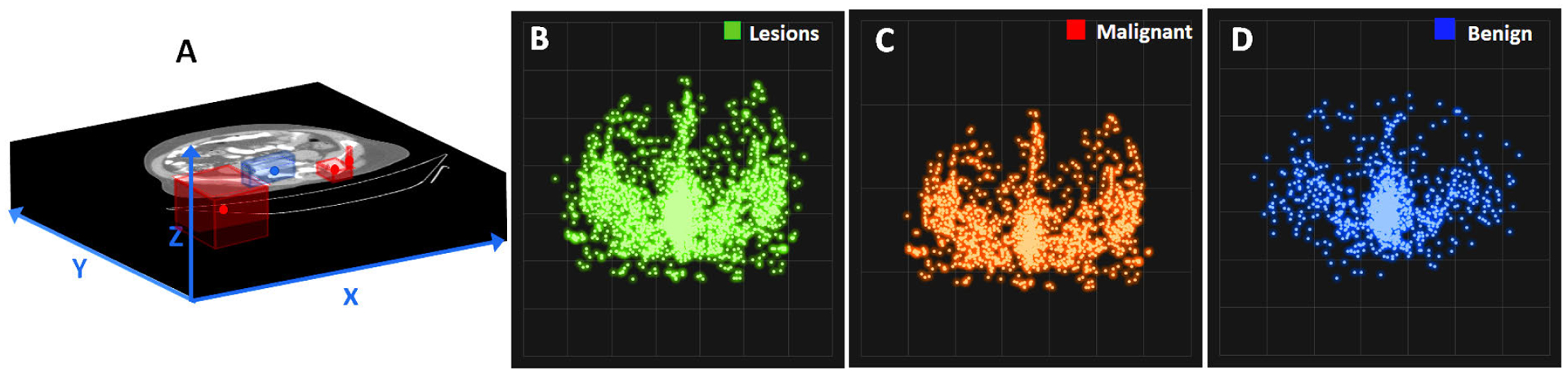
A) CT slice image containing a degenerative joint disease (DJD) benign lesion, and three sites of mixed and osteoblastic lesions, B) 2-D scatter plot of lesion centers mapped into 714 × 714 axial plane, and its equivalent for (C) malignant and (D) benign lesions.

**FIGURE 4.**
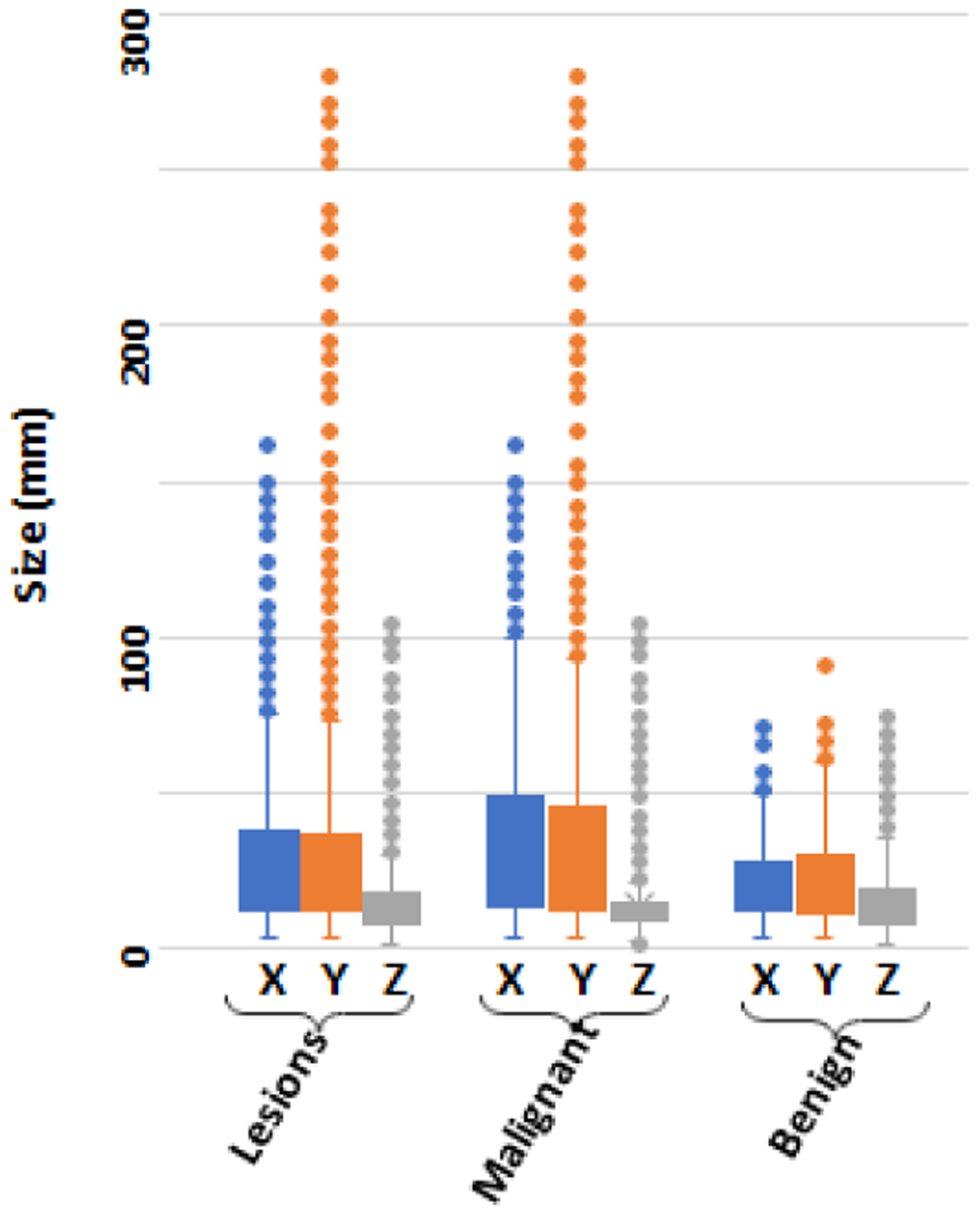
Lesions dimensions.

Clinical imaging for staging prostate cancer patients should ideally provide information on the status of the bone lesion. There are many critical factors that are known to affect the estimation of the tumor status. Such well-established factors include the destruction pattern in bone texture, characteristics of the lesion borders and its surrounding transition zone, presence of periosteal reaction, the location of the lesion (long or flat bone, appendicular or axial skeleton, epi-/meta-/ or diaphyseal, central or peripheral), the lesion extent and its growth rate, monostotic or multilocular occurrence, and patient age and ethnicity. Here, we provided the descriptive lesion statistics regarding their size and location in our dataset which helped us to better design our proposed solution (Table 1, Figures 3, and 4). Figure 3 shows how cancer lesions are widely spread around the bone structure in axial planes, while benign bone lesions (such as degenerative joint disease) are mostly focused around the vertebra. Depicted by Figure 4, larger lesions tend to be cancerous. Also, benign lesions seem to fall around a 1:1:1 XYZ aspect ratios compared to varying aspect ratios of cancer lesions. Thus, there is clear evidence that size and location can majorly contribute to the lesion type.

### C. CT SCAN PREPROCESSING

The CT images were re-sampled to have similar voxel spacing of .7mm × .7mm × 1mm in [x, y, z] and symmetrically zero-padded or cropped to the same size of 714 × 714 × 39 voxels. We used a bone-window to clip CT images and mapped their intensity values into 8-bit integer numbers.

### D. LESION-AWARE DATA STRATIFICATION

**FIGURE 5.**
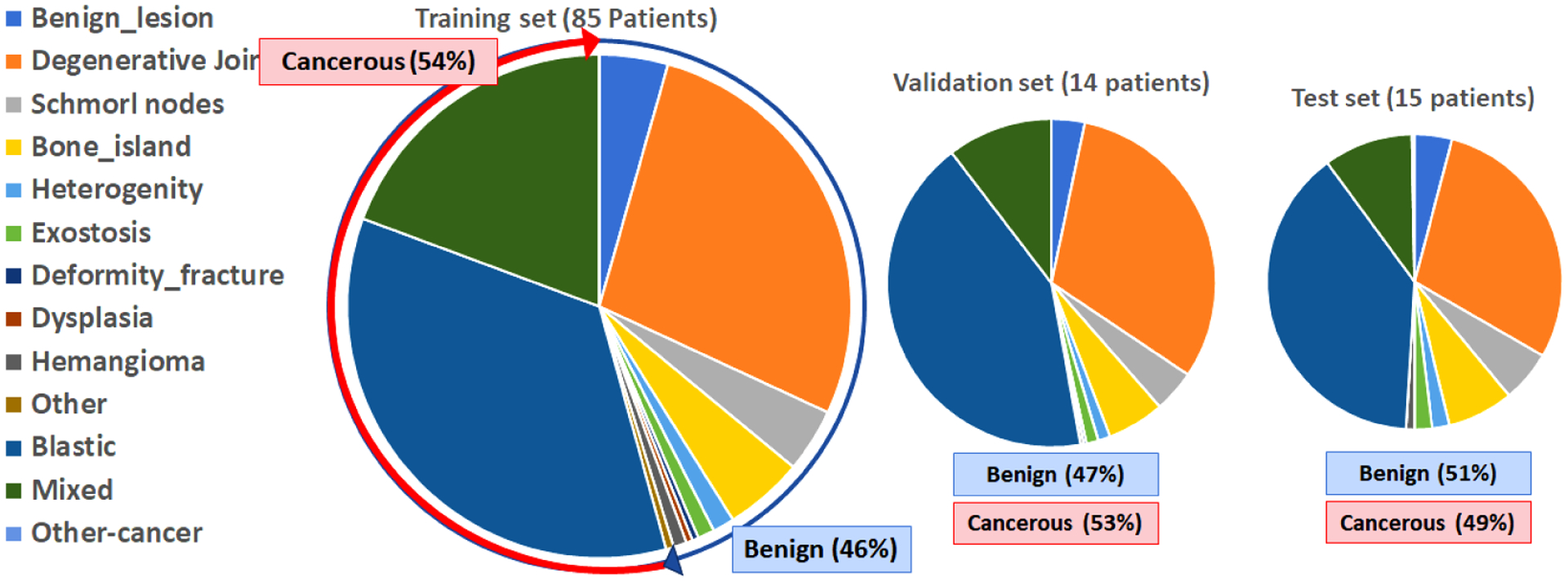
Patient-level lesion-aware data splitting based on equivalent distribution of lesion types.

In order to train and evaluate our binary classification algorithm, we divided our dataset into three splits of training (75%), validation (12%), and test (13%) at a patient-level which means all lesions from an individual patient are included in only one of the three splits. As was mentioned earlier, this dataset is limited to 114 patients with a variety of 13 lesion sub-categories from either major groups of benign or malignant. Our preliminary study of the bone lesions in prostate cancer patients [26], supported a theory that: “having a lesion-aware data stratification, can improve the generalizability of our lesion classification model”; Every lesion subcategory may introduce a certain set of features that must be generalized into two major groups by the network through a *deep* learning process. Insufficient number of samples from one sub-category for training may cause random or wrong decisions by the network during the inference and thus reduces the generalizability of the model. Instead of conventional approach for randomly splitting the patients, or just accounting for benign-malignant ratio, We used detailed descriptive information of the lesions to split our dataset. To construct a representative training set for bone lesion classification, we: 1) Performed a patient-level data stratification, 2) Kept similar balanced ratio of benign-malignant lesions within each split, 3) Incorporated lesions from very rare subcategories (less than 20 lesions of a kind in the whole dataset) for training only (we observed some lesion types like osteoblastic metastases or degenerative joint disease are far more prevalent than the others like fibrous dysplasia in our dataset), 4) Kept similar standardized distribution of lesion subcategories within each split. Table 1 and Figure 5 show the obtained distribution of lesion data in three splits. Details of stratification strategy are described in the Appendix B. The equivalent distribution of patches in three splits is also presented by Figure 12. Next, we designed several experiments to study the most effective elements in lesion classification based on lesion/patch extraction and the algorithm itself.

### E. LESION EXTRACTION

**FIGURE 6.**
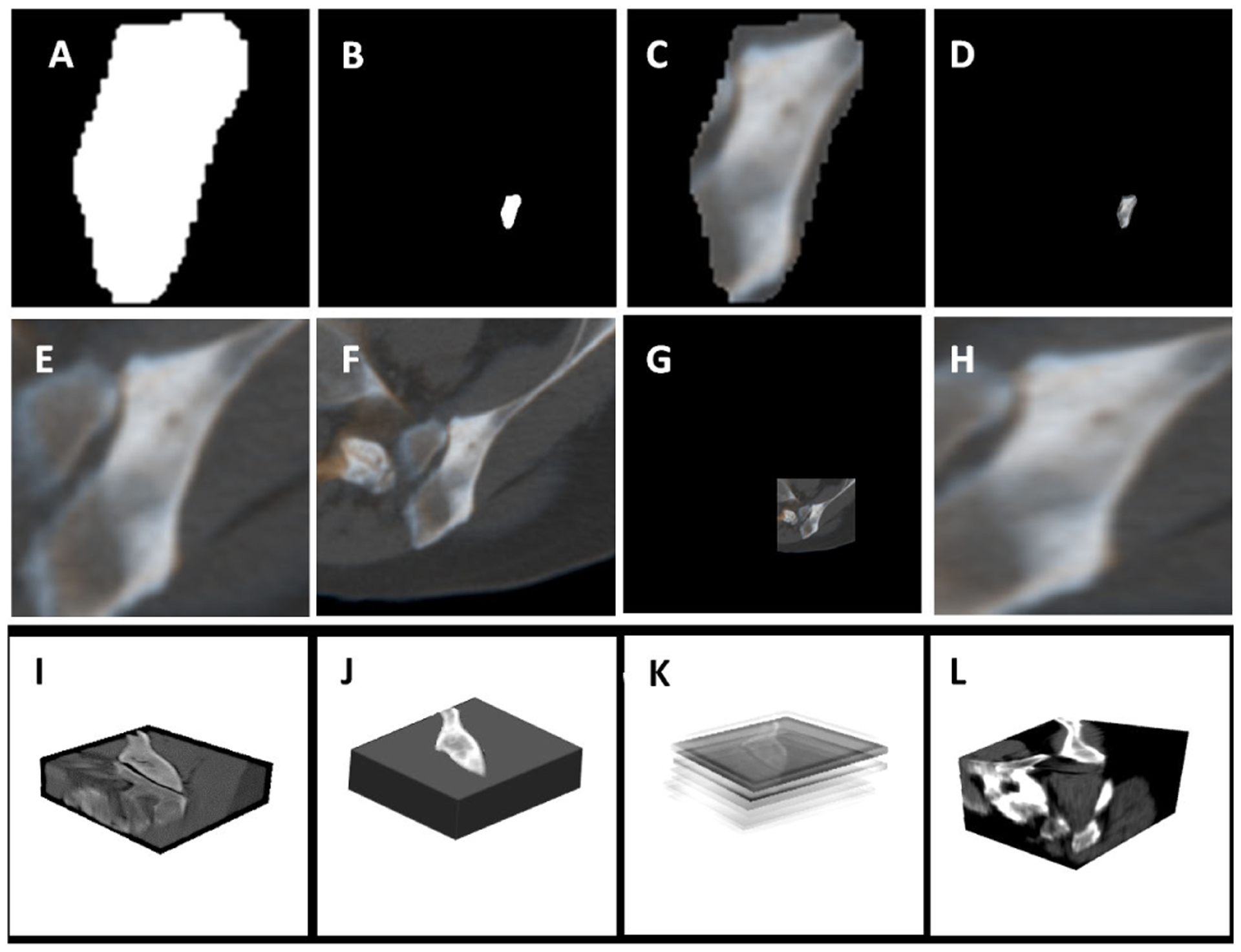
A mixed cancer lesion in 12 different patch extraction formats: A) lesion mask, B) lesion mask in its original location, C) masked lesion patch, D) masked lesion at its original location, E) original square dimension patch, F) square constant dimension patch, G) Square constant dimension patch at original location, H) original rectangular dimension patch, I) 145 × 145 × 39 constant dimension lesion, J) original masked lesion, K) sliding 145 × 145 × 7 lesion volume extraction, L) original size lesion.

A lesion can be extracted in various ways. A common way is to define a rectangular region of interest (ROI), and then use this as an input to the machine learning classifier. However, ROI definition may set boundaries to the lesion characteristics that can contribute to the classification accuracy. To comprehensively explore the effect of ROI definition, we employed 12 different patch and lesion extraction strategies. These strategies are designed to study the effect of different factors, such as size, morphology, X-Y dimension aspect ratio, lesion texture, location, and volumetric information. The first two rows in Figure 6, depict 8 patch extraction methods from CT images. Patches demonstrated in RGB colors represent the z-information, i.e. in addition to the main slice as the green channel, two immediate neighboring slices are incorporated as red and blue (2.5D which will be referred by A, B, …, and H strategies). Using the same patch extraction methods, we also experimented with 2D patches, that are extracted exclusively from the CT slice where the lesion occurred (which will be referred by A’, B’, …, and H’ strategies). Furthermore, we used 4 lesion extraction methods demonstrated by the last row in Figure 6. The specifications for all these patch and lesion extraction methods are listed in Tables 2, and 3. All patch extraction methods, except H, are based on a square shape to avoid losing the aspect ratio and morphological information while extracting the lesions in the axial plane. ROI delineation in methods B, D, and G are approximations to incorporate local information. To account for the lesion size, we used 173 × 173 pixels (99th percentile of the patch dimensions) to extract the patches. Additionally, we utilized the 95th percentile lesion dimension in both X and Y directions to extract 145 × 145 × 39 voxel lesions in method I. Method L implies extracting the lesion bounding volume, which resembles method H but in 3D. Method K, describes the use of sliding volumes of size 145 × 145 × 7 voxels with 145 × 145 × 5 voxel overlap sliding along the z direction.

### F. DATA AUGMENTATION

To augment 2D, 2.5D, and 3D images, we used random horizontal flipping (x-direction according to Figure 3.A), random zooming (within the range of [0.75, 1.25] of the original size), and random spatial rotation in an interval of (−15¤, +15¤) for lesion extraction. Additionally, we used random flipping in z-direction for both 2.5D and 3D data augmentation. Furthermore, to mimic and learn about the uncertainties that exists with lesion delineation by either radiologist or a detection algorithm we employed an *unfocused* lesion extraction for data augmentation. The details of this augmentation are provided in Appendix A which provides a realistic training set and thus results in a potentially more reliable model.

### G. CNN ARCHITECTURES

**FIGURE 7.**
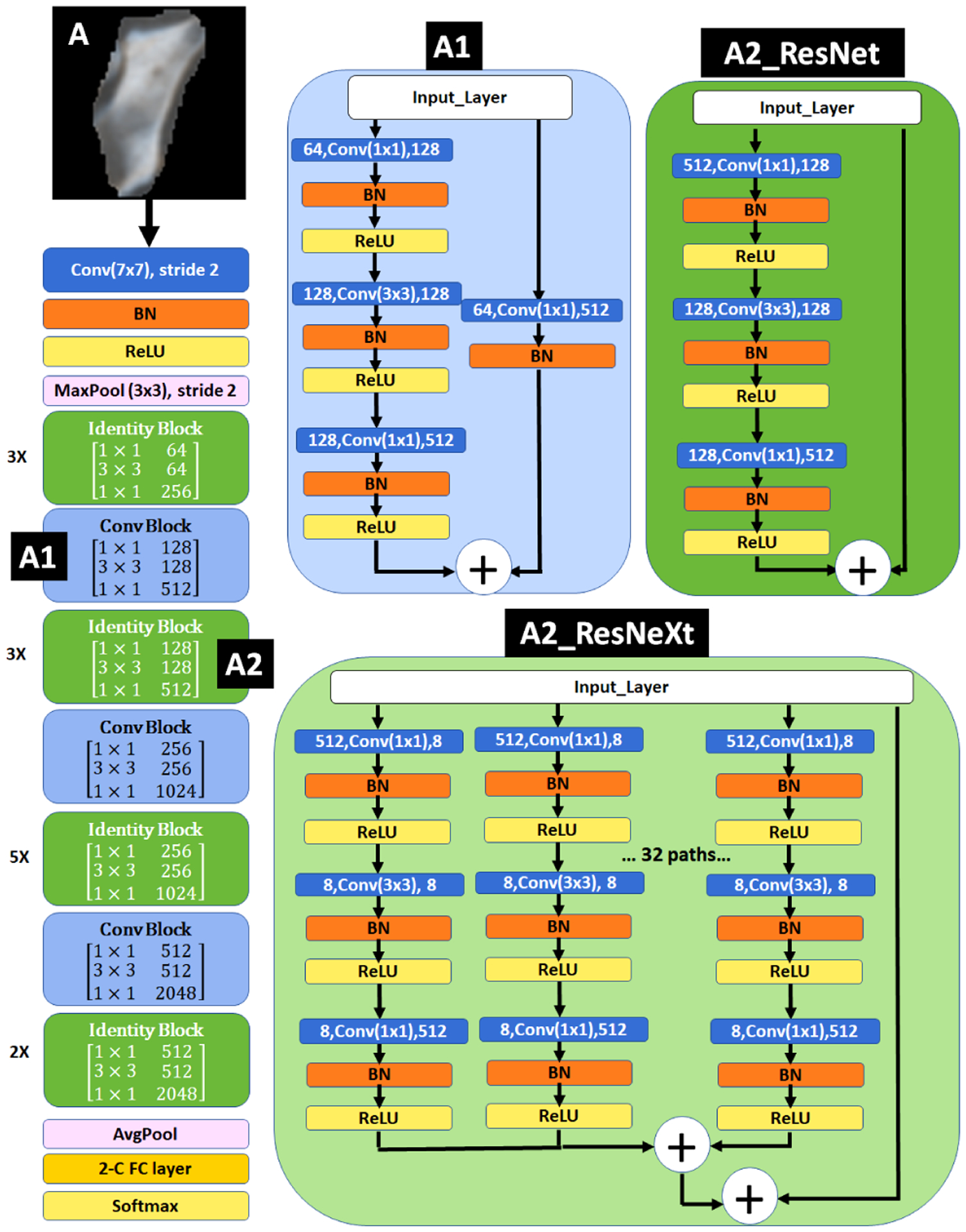
2D ResNet-50 with its first Conv Block represented as A1, and second Identity block represented as A2. The A2 identity block is replaced in ResNeXt-50 using 32 cardinality.

Our proposed solution for binary classification of bone lesions is composed of 3 stages: in **2D-analysis**, we employed the original architectures of 2D ResNet-50 and 2D ResNeXt-50 to train a classifier using 2D and 2.5D patches. To train each model, we minimized the binary cross-entropy loss function for 100 epochs, utilizing stochastic gradient descent (SGD) optimizer and initial learning rate of 1e^−3^ with 0.8 drop rate every 7 epochs. We chose binary cross-entropy as the loss function since it well fits our balanced binary classification problem. We utilized SGD rather than adaptive methods since SGD is able to generalize better (often significantly better) than the adaptive optimization methods even with their better training performance specifically for the classification task [27]. We also used a weight regularization of 2e^−4^ to avoid overfitting. Exploring ResNeXt-50 to address this task, we empirically found the cardinality of 32 to be the optimum for this purpose (Figure 7). While loss function and performance metrics imply patch-level training, we used a lesion-based average voting to draw a conclusion regarding the whole lesion during the inference. To do so, dual probability vectors generated by the last dense layer of the deep network for patches within a lesion are averaged to make a decision for that lesion.

**FIGURE 8.**
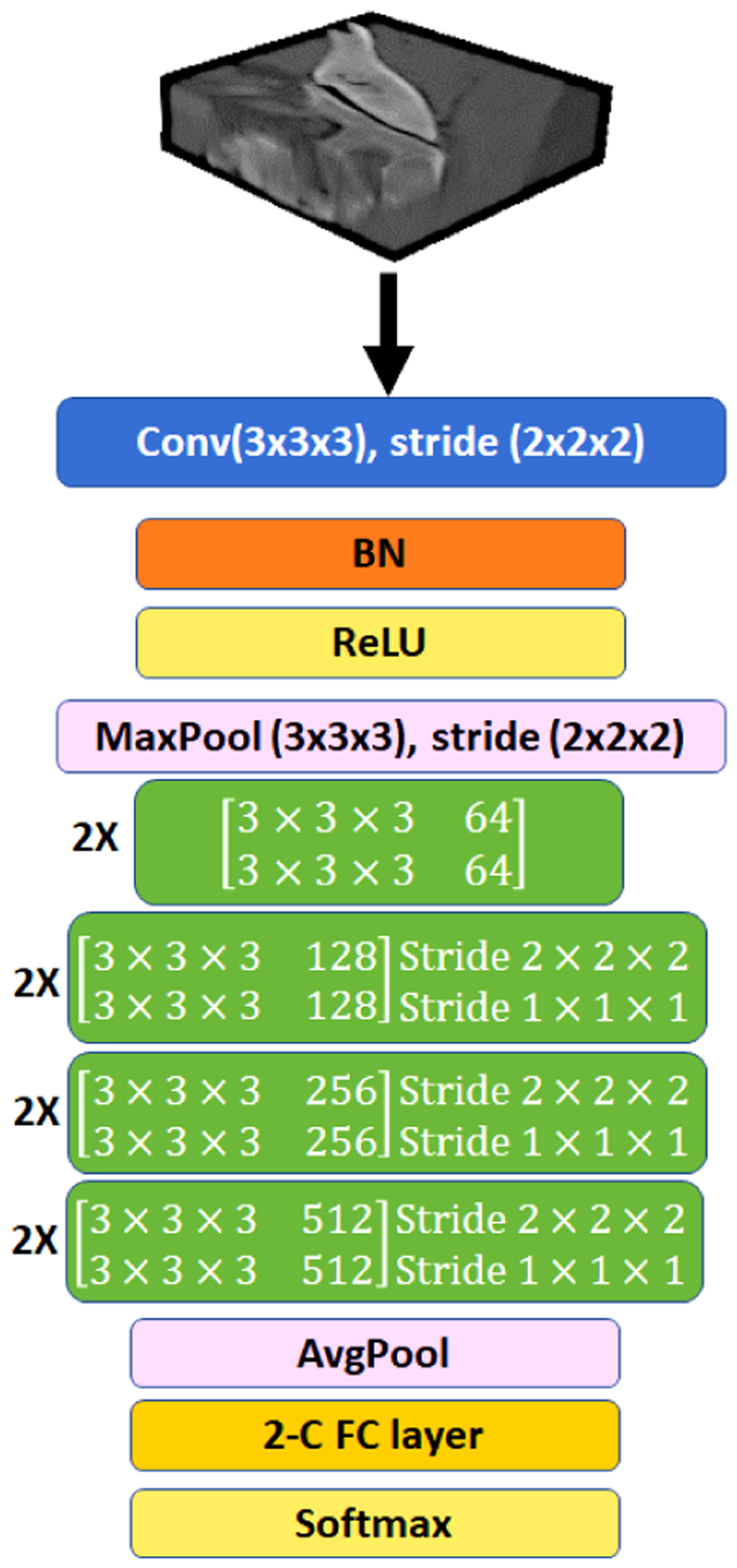
3D ResNet-18.

In another round of experiments for **3D-analysis**, we used 3D ResNet-18, and 3D ResNet-50 for lesion classification with 3× 3 × 3 convolutional layer as the first layer (Figure 8) since we did not have as many lesions in 3D for training which is discussed in Appendix C. Limited by the dataset, to avoid undertraining, we restricted ourselves to ResNet-18 with fewer parameters along with strategies I, J, and L (volumetric lesions). With at most 100 epochs of training for this model, we optimized its weights based on binary cross-entropy loss function. To do so we used an initial learning rate of 3e^−3^ with 0.85 drop rate every 5 epochs and an early stopping condition where training stops after 10 epochs of no improvement in validation loss.

**FIGURE 9.**
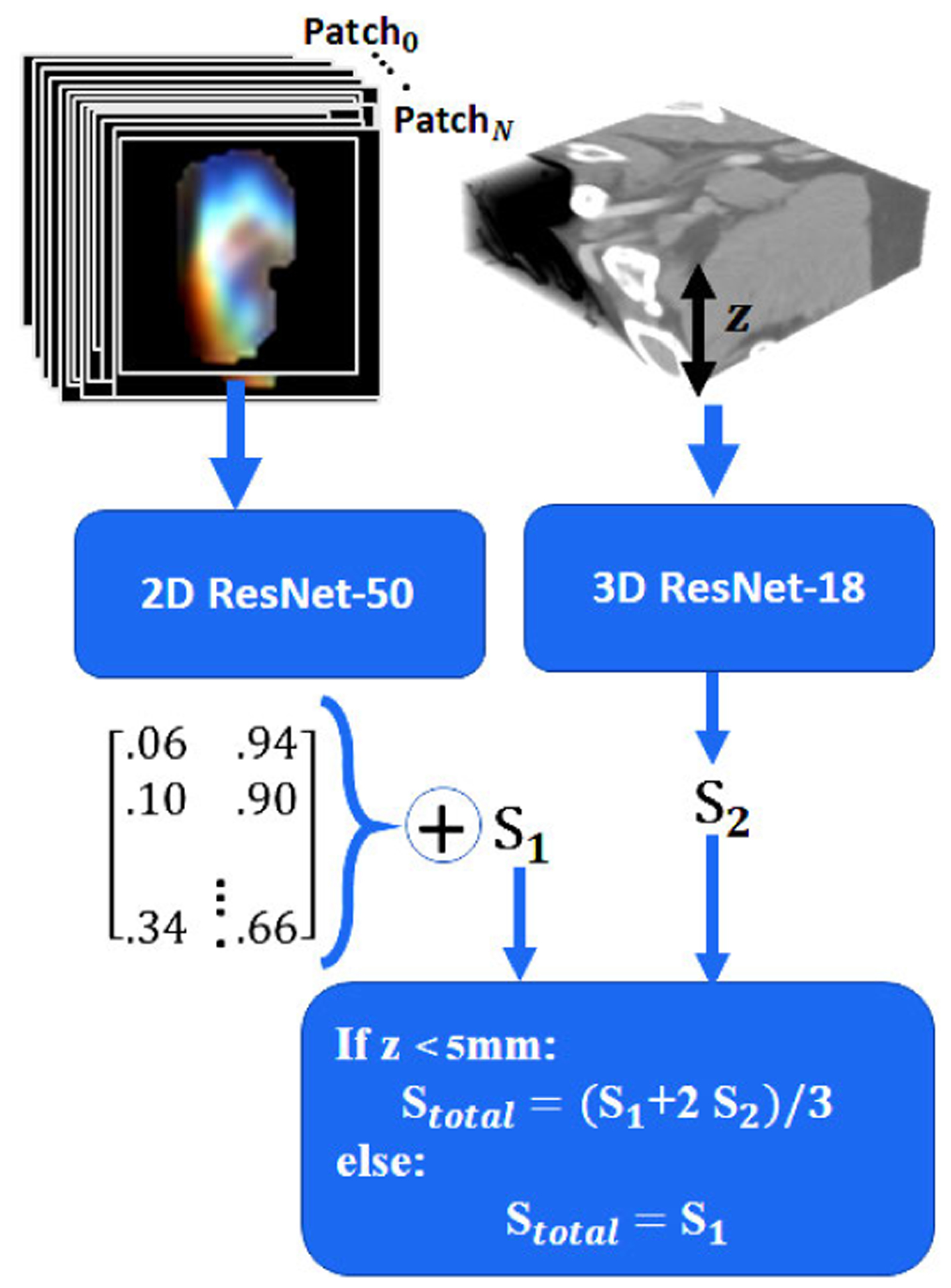
Ensemble of 2D ResNet-50 trained by strategy C and 3D ResNet-18 trained by strategy I for bone lesion classification.

**FIGURE 12.**
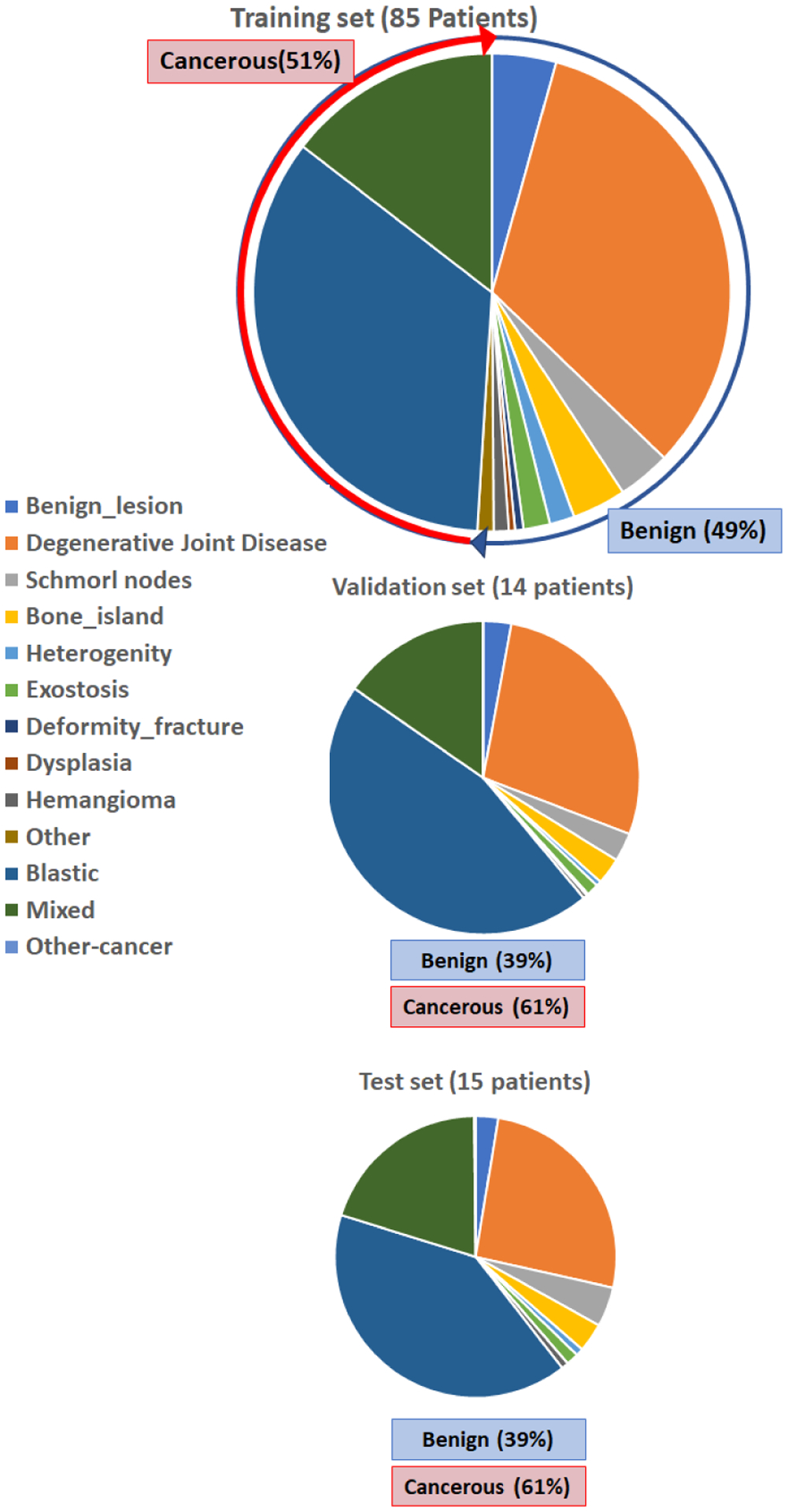
Equivalent 2D Patch distribution in 3 splits for training, validation, and test.

Next, we used 3D ResNet-50 to be trained by lesions extracted through method K (sliding volumes of 145 × 145 × 7 over a lesion) as this strategy multiplies the original data population by multiple times. We trained this 3D model similarly with an initial learning rate of 5e^−3^. Similar to 2D-analysis, a weighted average of the scores obtained for the sliding volumes was used to estimate the lesion category where marginal volumes weighted less in final calculations. To further improve upon these results, we eventually used a **policy-based ensemble** of lesion-based average voting 2D ResNet-50 utilizing strategy C (morphological texture patches) and 3D ResNet-18 utilizing strategy I (texture lesions). Our policy implies a 1:2 voting credit for 2D:3D models in case of smaller lesions (z < 5mm) and an exclusive reliance on the 2D model otherwise. Based on our preliminary evaluations, each of these two algorithms addresses the classification problem through a different perspective. The 2D ResNet-50 (trained with strategy C) is focused on characterizing the lesion texture at a patch-level and 3D ResNet-18 is concerned with an overall location, depth, and volumetric information rather than just the texture of the lesion (Figure 9).

## III. RESULTS

To evaluate our binary classification, we used accuracy (Acc) to describe correct classification of both benign and malignant lesions. Additionally, as false positives and false negatives are just equally important in this application, we measured misclassification using F1-score. We reported both performance metrics at the slice-level (SL) as well as the lesion-level (LL), and the patient-level (PL). Slice-level results imply the algorithm decision for each slice while lesion-level outcomes are computed based on the majority vote of the slices contained within each lesion. To transform the lesion-level results into patient-level, we used a condition where having 3 metastatic lesions per patient or 20% metastatic lesions per patient implies being metastatic. The results of Experiment-1, 2.5D classification with ResNet-50 on the validation and test splits are respectively presented by Tables 4 and 6. Employing strategies C, E, F, and H on the validation set (Table 4), showed higher comparable lesion-level accuracies and F1-scores among the others. However, the ResNet-50 model trained with strategy C patches (using texture and morphology), resulted in the fewest missed cancerous lesions per patient (1.2 lesions per patient). In Experiment-2 we compared the performance of ResNet-50 when trained using 2D patches as opposed to Experiment-1 with 2.5D patches. In this experiment, we used five strategies (C, D, E, F, and H) for 2D patch extraction to train the ResNet-50 classifier. The performance metrics for validation and test splits are reported by Table 5, and 6 respectively. Comparing Experiments-1 and −2 in Tables 4 and 5, as well as the test results in Table 6, one can notice superior or equal lesion-level performance when using 2.5D patches against 2D patches with the exception for strategy D. Such improvement in performance is due to the partial incorporation of the volumetric information by 2.5D patches. With strategy D, which was designed to include all types of lesion characteristics such as XY location, size, texture and morphology, ResNet-50 failed to efficiently utilize this information from 2.5D patches. We believe that switching from 2.5D to 2D, by excluding volumetric information from strategy D, we could facilitate learning and improve the performance in terms of both accuracy and F1-score.

To explore the potential improvement in our classification results with ResNeXt-50, we arranged Experiment-3 using 2.5D patch extraction strategies C, E, and D. After training and validation, we compared the results of ResNeXt-50 models against ResNet-50 on the test split (Table 7). With the slice-level improvement in more complex strategies C and D, lesion-level performances are comparable in all cases. Bringing cardinality as another hyperparameter into account, has leveraged ResNeXt-50 in decoding different lesion characteristics that are contained within patches of strategy D [6]. We have also included the equivalent results for validation in the Appendix C. Investigating all the results obtained in Experiments-1, −2, and −3, one can see that lesion-aware data stratification could help our 2D models to generalize during the training and validation, achieving the same or even better performance on the test set. Also, confirmed unanimously by the results from all these experiments, employing strategy C for patch extraction could escalate the learning capability using ResNet-50 and ResNeXt-50. More importantly, we could observe that texture is the most significant factor that determines the classification results. Last but not least, morphology by itself proved useful with lesion categorization supported by Experiment-1 using strategies A and B.

On the other hand, and unlike what could be theoretically expected, defining the ROI with the additional information about lesion location has downgraded the results. According to all three Experiments-1, −2, and −3, applying algorithms with certain capacity to B, D or G patch extraction strategies with larger patches (714 × 714) practically fails in comparison to smaller patches (176 × 176 pixel). The reason for this failure, could be ResNet-50 receiving less textural or morphological information at the expense of lesion location. In fact, thorough information on lesion texture solely, has a more significant role than the location. We also learned that background information interferes with the decision system by complicating the training, once we compared the algorithms performance using strategies C and F. We have further discussed the sufficiency of ResNet-50 against a larger model such as ResNet-101 in the Appendix D.

In our 3D Experiment-4, we applied 3D ResNet-18 to process 3D lesion information for classification using strategies I, J, and H. We dedicated Experiment-5 to train the 3D ResNet-50 classifier using the lesions extracted by strategy K. The corresponding results are shown in Tables 8 and 9 for validation and test sets, respectively. Sufficiency of these two models for these tasks are discussed in Appendix E. Confirmed by the obtained performance metrics, morphology is a less effective, or even crippling factor in a 3D sense. Overall, results of Experiment-5 (3D ResNet-50 applied to lesions extracted by strategy K, sliding volumes) outperformed the results from Experiment-4. However, 3D ResNet-18 proved to be an efficient method, when applied to the lesions obtained using strategy I (whole volume). This approach demonstrates a totally different performance in terms of its predictions when compared to Experiment-1 (2D ResNet-50 applied to 2.5D patches). Supported by our preliminary evaluations [26] and the results obtained in this work, we believe 2D and 3D methods learn to base their predictions on a totally different set of the features. In our final study, Experiment-6, we exploited this contrasting performance through a policy-based ensemble method incorporating the methodologies in Experiment-1 with strategy C and Experiemnt-4 with strategy I. The results for such an ensemble approach are highlighted against each individual method in Table 10. Performance measures in this table confirm the achieved improvement using the ensemble method during both validation and test.

## IV. CONCLUSION

Lesion categorization is an inevitable element of fully automated lesion detection algorithms and is generally too challenging to be addressed in the detection step due to the overlapping appearance of the bone lesion categories. Suggested by the literature [22]–[26], [28]–[30], most lesion detection algorithms fail to provide a high recall with an acceptable false positive rate in a single pass. On the other hand, a highly accurate classification algorithm is always desired since miss-classification can negatively impact the patient’s quality of life. Thus, the state-of-the-art in this field relies on cascading at least two deep learning algorithms; automatic detection followed by deep classification algorithm. Here, we focused on the second part of this problem. For this purpose, we gathered a great number of metastatic and benign bone lesions from prostate cancer patients, carefully annotated by an expert radiologist. We curated our data by appropriate preprocessing, matched their spacing, and zero-padded them to achieve the same size in axial plane. To enhance the generalizability from training for our classification algorithm, we proposed to split our patients into three groups following the same lesion subcategory distribution. Additionally, to better understand the impact of ROI-introduced factors that incorporate in the training process of our classifier, we designed 12 different lesion extraction strategies. We employed four different state-of-the-art deep classifiers utilizing different patch/lesion extraction strategies to develop an optimal model for bone lesion categorization. Through 6 different experiments, we investigated which factors are learn-able by such deep neural network architectures and measured their impact on bone lesion classification. For better training, we applied various augmentation schemes plus unfocused patch/lesion extraction to mimic the real-word lesion extraction scenarios. Our evaluations showed that: (1) texture, as expected, is the most informative part of a lesion image, (2) volumetric information is the second leading factor by introducing a totally different set of the features for this task, and (3) lesion morphology has a limited role in lesion classification, due to the performance of the algorithms utilizing patches from strategy A, B, and C. We also discussed how incorporating local information had negatively affected the importance of patch texture and morphology, and thus decreased the overall accuracy. A substitute solution to avoid this negative effect, could be to append the regressed version of lesion coordinates directly to the last dense layer of the classifier. Theoretically, such a method could preserve texture and morphology while having local clues for better classification, however, it requires a pre-skeletal registration which was beyond the scope of this paper. We used ResNeXt-50 in Experiment-2 with cardinality of 32 for 2.5D patch classification which proved to perform better than ResNet-50 at the slice level and with similar performance at a lesion level. ResNeXt-50 could significantly improve the performance with strategy D by decomposing the input elements for better training. To avoid under-training in 3D we employed 3D ResNet-18, and 3D ResNet-50 in Experiments-4, and −5 with the best results using lesion extraction strategies I and K. With the switch from 2.5D to 3D, we experienced a drop in results caused by the absence of lesion-based average voting and a larger number of parameters to be optimized, accompanied by fewer training examples in 3D. While the results from 3D-analysis are less significant compared to 2D-analysis, they present a different perspective for bone lesion evaluation. Thus, we combined the 2D ResNet-50 trained by 2.5D patches from strategy C with 3D ResNet-18 trained by lesion extracted using strategy I to bring in different perspectives together for more reliability in lesion classification. In the future, we will cascade this proposed AI classification method to a bone lesion detection algorithm that enables a fully automated detection and classification approach to potentially improve the radiologist performance when interpreting CT scans in prostate cancer patients.

## Figures and Tables

**FIGURE 10.**
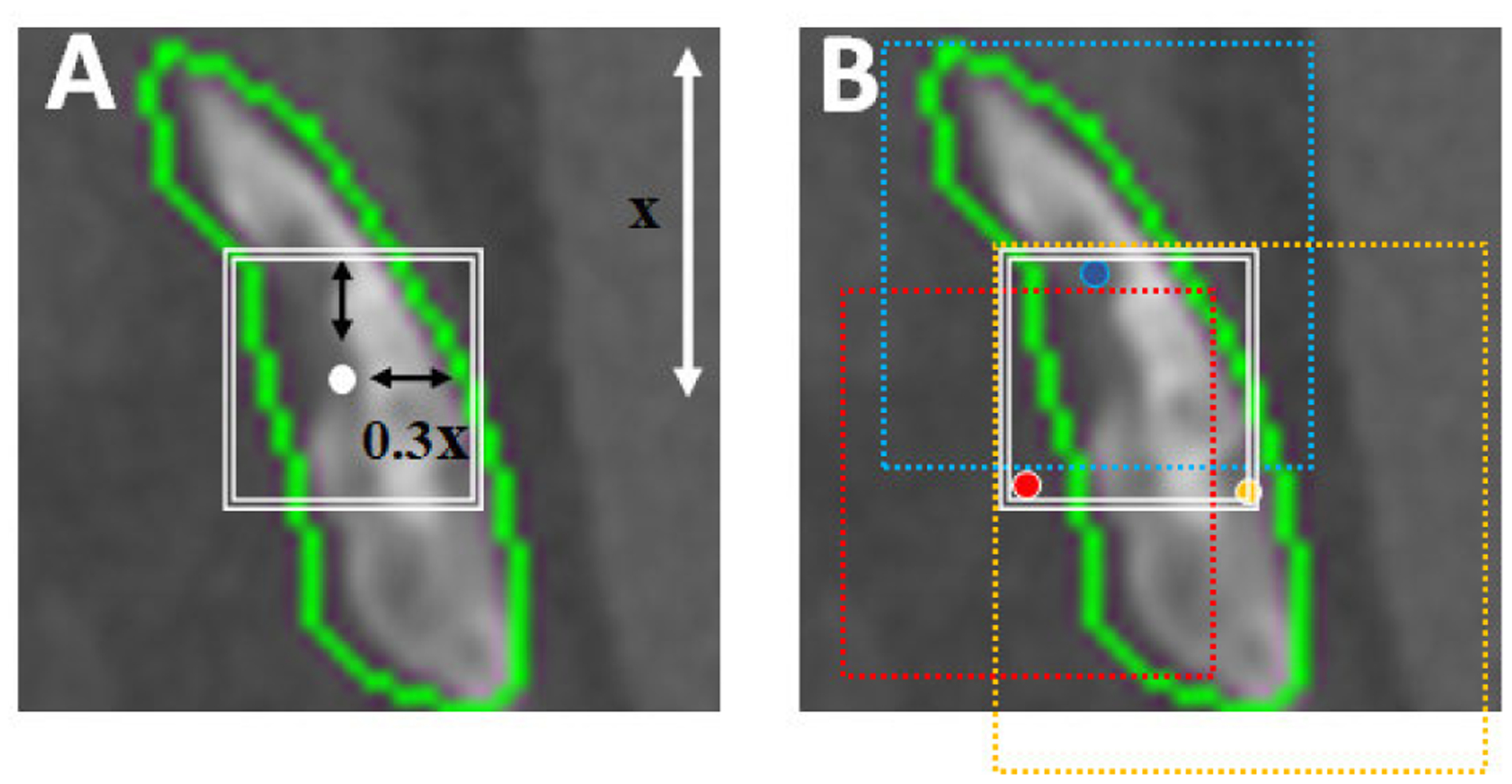
Unfocused lesion extraction for augmentation.

**FIGURE 11.**
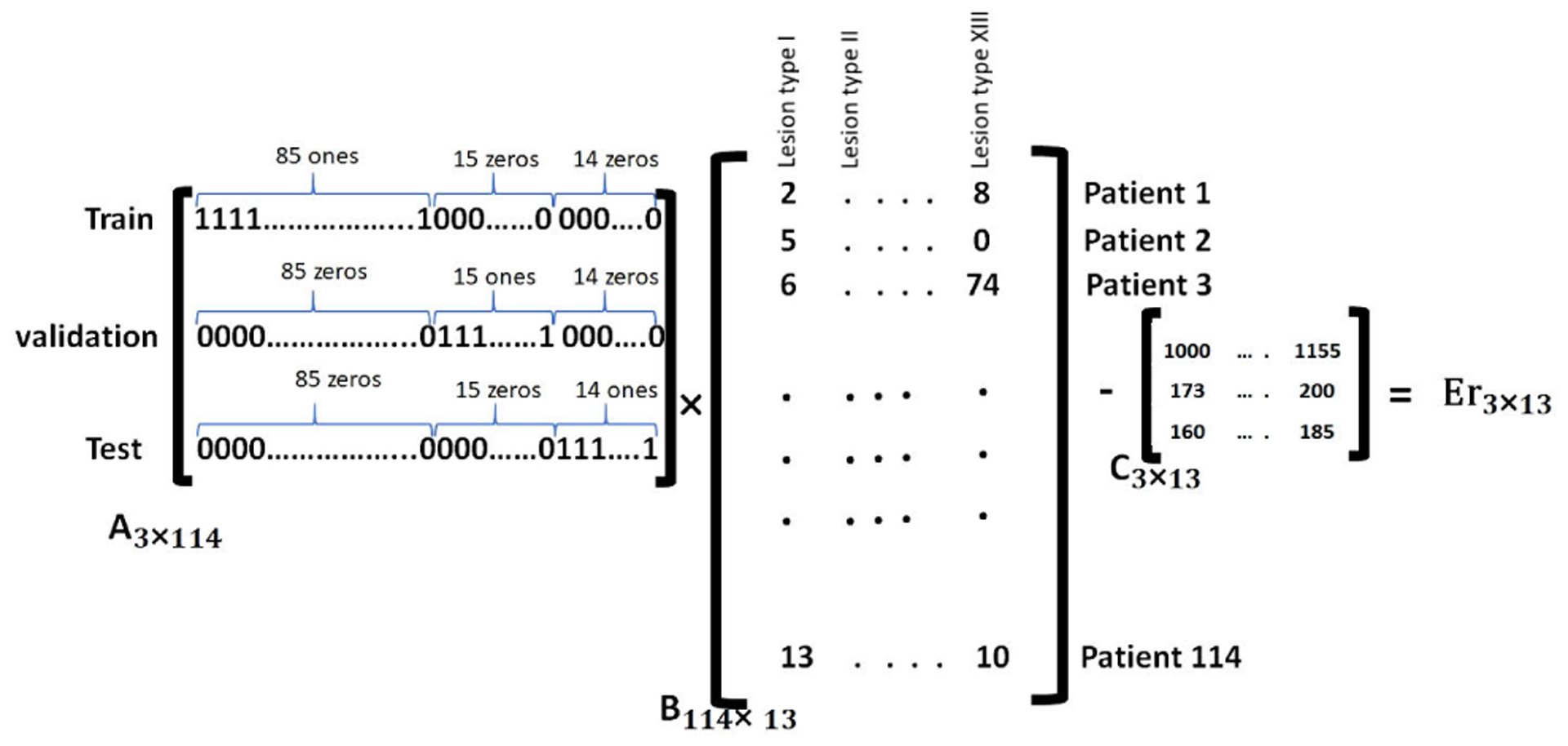
Linear equations used to distribute lesions into three splits at a patient-level with equivalent distribution of various lesion types.

**TABLE 1.** Data statistics (*Total and Per-Patient*).

Split	Patient	Total Lesions *Per Patient*	Malignant *Per Patient*	Benign *Per Patient*
**Train**	85	2225	1207	1018
		26.2±59.3	14.2±60.9	12±8.1
**Valid**	14	337	178	159
		24±17.9	12.7±17.6	11.4±6
**Test**	15	318	156	162
		21.2±19.1	10.4±18	10.8±7.8
**Total**	114	2880	1541	1339
		25.3±52	13.5±53.4	11.7±7.8

**TABLE 2.** Patch extraction strategy description.

Patch Extraction	Size	Morphology	Texture	Location
A		⨯		
B		⨯		⨯
C		⨯	⨯	
D	⨯	⨯	⨯	⨯
E			⨯	
F	⨯		⨯	
G	⨯		⨯	⨯
H			⨯*	

**TABLE 3.** Lesion extraction strategy description.

Lesion Extraction	Size	Morphology	Texture
**I**		**⨯**	
**J**		**⨯**	
**K** (sliding volume over the lesion)		**⨯**	**⨯**
**L**	**⨯**	**⨯**	**⨯**

**TABLE 4.** Validation results of Experiment-1, ResNet-50 using 2.5D patch extraction strategies at slice-level (SL), lesion-level (LL), patient-level (PL).

Strategy	Acc (SL)	F1-Score (SL)	Acc (LL)	F1-Score (LL)	Acc (PL)	F1-Score (PL)
**A**	61.6%	63.1%	65.9%	67.4%	78.6%	87.0%
**B**	58.0%	51.7%	59.9%	47.9%	64.3%	70.6%
**C**	80.5%	80.6%	86.4%	86.3%	**92.9%**	**94.7%**
**D**	66.3%	62.8%	70.3%	65.5%	78.6%	84.2%
**E**	82.7%	83.4%	**88.4%**	**88.9%**	85.7%	88.9%
**F**	76.4%	76.3%	81.0%	81.0%	78.6%	84.2%
**G**	70.8%	69.1%	73.3%	71.9%	64.3%	71.4%
**H**	**85.1%**	**86.3%**	85.5%	87.0%	78.6%	85.7%

**TABLE 5.** Validation results of Experiment-2, ResNet-50 using 2D patch extraction strategies at slice-level (SL), lesion-level (LL).

Strategy	Acc (SL)	F1-Score (SL)	Acc (LL)	F1-Score (LL)
**C’**	83.3%	84.6%	86.1%	87.7%
**D’**	71.1%	70.3%	75.1%	74.9%
**E’**	81.9%	82.9%	86.4%	87.7%
**F’**	78.5%	79.7%	80.4%	81.6%
**H’**	83.4%	84.4%	87.8%	89.1%

**TABLE 6.** Test results of Experiment-1, −2, ResNet-50 with 2D & 2.5D patch extraction strategies at slice-level (SL), lesion-level (LL), patient-level (PL).

Strategy	Acc (SL)	F1-Score (SL)	Acc (LL)	F1-Score (LL)	Acc (PL)	F1-Score (PL)
**C**	84.4%	85.9%	**91.2%**	**91.1%**	**100.0%**	**100.0%**
**E**	83.5%	84.5%	**89.6%**	**90.5%**	93.3%	90.9%
**F**	80.5%	82.0%	82.4%	81.5%	100.0%	100.0%
**H**	**86.0%**	**88.2%**	84.6%	86.1%	80.0%	76.9%
**C’**	84.1%	86.0%	89.6%	89.5%	100.0%	100.0%
**D’**	69.9%	71.3%	69.5%	66.7%	73.3%	71.4%
**E’**	83.5%	85.3%	88.4%	88.6%	100.0%	100.0%
**F’**	78.2%	79.7%	81.1%	80.0%	93.3%	90.9%
**H’**	81.8%	83.5%	86.2%	85.2%	100.0%	100.0%

**TABLE 7.** Test results of Experiemnt-1, −3, ResNet-50 and ResNeXt-50 using 2.5D patch extraction strategies at slice-level (SL), lesion-level (LL), patient-level (PL).

Method	Acc (SL)	F1-Score (SL)	Acc (LL)	F1-Score (LL)	Acc (PL)	F1-Score (PL)
**ResNet50-C**	84.4%	85.9%	**91.2%**	**91.1%**	**100.0%**	**100.0%**
**ResNet50-E**	83.5%	84.5%	**89.6%**	**90.5%**	92.9%	94.7%
**ResNet50-D**	71.1%	70.8%	76.4%	72.3%	80.0%	72.7%
**ResNeXt50-C**	**85.2%**	**87.3%**	90.5%	90.9%	100.0%	100.0%
**ResNeXt50-E**	81.0%	83.2%	82.4%	82.2%	93.3%	90.9%
**ResNeXt50-D**	74.4%	76.0%	76.7%	75.2%	93.3%	90.9%

**TABLE 8.** Validation results of Experiments-4, and −5, 3D ResNet-18, and 3D ResNet-50 methods using 3D lesion extraction strategies at slice-level (SL), lesion-level (LL), and patient-level (PL).

Strategy	Acc (LL)	F1-Score (LL)	Acc (PL)	F1-Score (PL)
**3D ResNet-18 (I)**	**79.4%**	**77.5%**	71.4%	77.8%
**3D ResNet-18 (J)**	77.1%	73.45%	71.4%	77.8%
**3D ResNet-50 (K) (K: Sliding Volumes)**	77.2%	**75.7%**	**85.7%**	**88.9%**
**3D ResNet-18 (H)**	78.9%	75.1%	85.7%	88.9%

**TABLE 9.** Test results of experiments-4, and −5, 3D ResNet-18, and 3D ResNet-50 methods using 3D lesion extraction strategies at slice-level (SL), lesion-level (LL), and patient-level (PL).

Strategy	Acc (LL)	F1-Score (LL)	Acc (PL)	F1-Score (PL)
**3D ResNet-18 (I)**	81.5%	**84.0%**	**93.3%**	**90.91%**
**3D ResNet-18 (J)**	77.8%	72.4%	93.3%	90.91%
**3D ResNet-50 (K) (K: Sliding Volumes)**	**84.6%**	83.3%	**93.3%**	**90.91%**
**3D ResNet-18 (H)**	79.9%	77.3%	93.3%	90.91%

**TABLE 10.** Results of Experiment-1 (2D ResNet-50 with stratgey C), Experiment-4 (3D ResNet-18 using strategy I), and Experiment-6 (ensemble of 2.5D and 3D analysis).

Method	PHASE	Acc (LL)	F1-Score (LL)	Acc (PL)	F1-Score (PL)
**3D ResNet-18 (I)**	VAL	79.4%	77.5%	71.4%	77.8%
	TEST	81.5%	84.0%	93.3%	90.91%
**2D ResNet-50 (C)**	VAL	86.4%	86.3%	92.9%	94.7%
	TEST	91.2%	91.1%	100.0%	100.0%
**ENSEMLE OF 2D and 3D models**	VAL	**87.0%**	**87.4%**	**92.9%**	**94.7%**
	TEST	**92.2%**	**92.3%**	**100.0%**	**100.0%**

**TABLE 11.** Validation results of Experiment-3, ResNeXt-50, using 2.5D patch extraction strategies at slice-level (SL), lesion-level (LL), patient-level (PL).

Method	Acc (SL)	F1-Score (SL)	Acc (LL)	F1-Score (LL)	Acc (PL)	F1-Score (PL)
**ResNeXt50-C**	79.0%	81.1%	84.0%	84.5%	78.6%	85.7%
**ResNeXt50-E**	81.9%	83.4%	84.3%	85.6%	78.6%	85.7%
**ResNeXt50-D**	73.7%	73.8%	77.2%	77.0%	85.7%	88.9%

**TABLE 12.** Validation results of 2D ResNet-50, and 2D ResNet-101 using different 2.5D patch extraction strategies at slice-level (SL), lesion-level(LL), and patient-level (PL).

Method	Acc (SL)	F1-Score (SL)	Acc (LL)	F1-Score (LL)	Acc (PL)	F1-Score (PL)
**ResNet50-C**	80.5%	80.6%	86.4%	86.3%	**92.9%**	**94.7%**
**ResNet50-E**	82.7%	83.4%	**88.4%**	**88.9%**	85.7%	88.9%
**ResNet50-F**	76.4%	76.3%	81.0%	81.0%	78.6%	84.2%
**ResNet101-C**	**84.2%**	**85.4%**	86.4%	86.7%	92.9%	94.7%
**ResNet101-E**	80.7%	80.5%	82.2%	81.0%	85.7%	88.9%
**ResNet101-F**	77.9%	79.1%	83.4%	83.1%	85.7%	88.9%

**TABLE 13.** Test results of 2D ResNet-50, and 2D ResNet-101 using different 2.5D patch extraction strategies at slice-level (SL), lesion-level(LL), and patient-level (PL).

Method	Acc (SL)	F1-Score (SL)	Acc (LL)	F1-Score (LL)	Acc (PL)	F1-Score (PL)
**ResNet50-C**	84.4%	85.9%	**91.2%**	**91.1%**	**100.00%**	**100.0%**
**ResNet50-E**	83.5%	84.5%	**89.6%**	**90.5%**	92.9%	94.7%
**ResNet50-F**	80.5%	82.0%	82.4%	81.5%	100.0%	100.0%
**ResNet101-C**	**86.3%**	**88.3%**	88.0%	89.3%	86.7%	83.3%
**ResNet101-E**	82.8%	84.9%	85.5%	84.5%	93.3%	90.9%
**ResNet101-F**	78.2%	80.2%	82.4%	80.8%	100.0%	100.0%

**TABLE 14.** Test and validation results of 3D ResNet-50, and 3D ResNet-34 using different lesion extraction strategies at lesion-level (LL).

	Validation		Test	
Method	Acc	F1-Score	Acc	F1-Score
**ResNet50-I**	61.6%	46.5%	62.3%	43.6%
**ResNet50-J**	65.3%	57.8%	59.1%	47.7%
**ResNet34-K**	75.7%	74.8%	80.2%	79.2%

**TABLE 15.** Confusion matrices for all major experiments where binary classification as benign or malignant correspond to negative or positive predictions, respectively. Thus, true negatives, true positives, and false negatives respectively impliy lesions truly classified as benign, truly classified as malignant, and falsely classified as benign which are reported at slice-level (SL), lesion-level (LL), and patient-level (PL).

Experiment	Method	Strategy	SPLIT	TNs (SL)	FNs (SL)	TPS (SL)	FPs (SL)	TNs (LL)	FNs (LL)	TPs (LL)	FPs (LL)	TNs (PL)	FNs (PL)	TPs (PL)	FPs (PL)
**Experiment-1**	**ResNet-50 (2D)**	C	VAL	2123	925	2150	110	146	33	145	13	4	1	9	0
			TEST	2015	711	2595	141	147	13	143	15	10	0	5	0
		D	VAL	2009	1565	1510	224	142	83	95	17	3	2	8	1
			TEST	1969	1392	1914	187	145	58	98	17	8	1	4	2
		E	VAL	2084	769	2306	149	141	21	157	18	4	2	8	0
			TEST	2104	849	2457	52	133	4	152	28	9	0	5	1
		F	VAL	2038	1098	2017	195	136	41	137	23	3	2	8	1
			TEST	1971	879	2426	185	139	33	123	23	10	0	5	0
		H	VAL	2026	524	2491	207	124	14	164	35	2	1	9	2
			TEST	1839	448	2858	317	117	4	152	45	7	0	5	3
**Experiment-2**	**ResNet-50 (2D)**	C’	VAL	1987	640	2435	246	153	29	149	6	3	0	10	1
			TEST	1927	639	2667	229	118	0	156	44	10	0	5	0
		D’	VAL	1959	1260	1815	274	131	75	103	28	4	1	9	0
			TEST	1776	1264	2042	380	121	38	118	41	6	0	5	4
		E’	VAL	2018	736	2329	215	147	26	152	12	2	2	8	2
			TEST	1946	691	2615	210	122	3	153	40	10	9	5	0
		F’	VAL	1927	835	2240	306	145	60	128	14	3	1	9	1
			TEST	1934	969	2337	222	118	18	138	44	9	0	5	1
		H’	VAL	2043	691	2384	190	155	43	135	4	3	0	10	1
			TEST	1953	791	2515	203	123	0	156	39	10	0	5	0
**Experiment-3**	**ResNeXt-50 (2D)**	C	VAL	1801	683	2392	432	136	31	147	23	2	1	9	2
			TEST	1876	528	2778	280	138	6	150	24	10	0	5	0
		E	VAL	1934	662	2413	299	127	21	157	32	2	1	9	2
			TEST	1854	736	2570	302	133	27	129	29	9	0	5	1
		D	VAL	1946	1109	1966	287	131	49	129	28	4	2	8	0
			TEST	1851	1093	2213	305	132	44	112	27	9	0	5	1
**Experiment-4**	**ResNet-18 (3D)**	I	VAL					149	59	119	10	3	3	7	1
			TEST					104	1	155	58	9	0	5	1
		J	VAL					153	71	107	6	3	3	7	1
			TEST					154	63	93	8	9	0	5	1
		H	VAL					159	71	107	0	4	2	8	0
			TEST					145	47	109	17	9	0	5	1
**Experiment-5**	**ResNet-50 (3D)**	K	VAL					140	48	120	19	4	2	8	0
			TEST					147	34	122	15	9	0	5	1
**Experiment-6**	**Ensemble (2D & 3D)**	C & I	VAL					140	25	153	19	4	1	9	0
			TEST					143	6	150	19	10	0	5	0

## APPENDIX A

### UNFOCUSED LESION EXTRACTION

To mimic the uncertainties that exits in real scenario of lesions extraction by either radiologist or the detection algorithm, we used unfocused lesion extraction to augment our dataset and enable the model to learn about these instabilities, and hence be more reliable (Figure 10).

In order to do so, we randomly chose a few spots within the 0.09 inner rectangular area of each lesion. We used these randomly chosen spots as the center for augmented lesions with their dimension to be within 1 ±.35 times the original lesion dimension. Prior to be considered for training, these lesions were investigated to ensure that they contain at least 50% of the lesion segmented area.

## APPENDIX B

### DATA STRATIFICATION

We believe that a generalized training set for a classification model demands: 1) patient-level data stratification, 2) balanced and preferably equal benign-malignant ratio within each split, 3) incorporation of rare lesions in training only, and 4) learning about different lesion subcategories following similar standard lesion distribution within each split. We managed to impose all 4 of the above-mentioned constraints into our data stratification using a simple search algorithm that minimizes the weighted average error of Er_3×13_ obtained by the equations from Figure 11. Overall, there were 13 comm lesion subcategories (considering very rare types in one category) in our dataset. We formed a matrix B_114×13_ representing the number of lesions from each subcategory for each patient. The assignment matrix A_3×114_ with 0/1 elements implies which patient is assigned to each of the three splits. Inherently, the sum of the elements in each column of A must equal 1, and sum of the elements in each of its rows is the number of patients included in each split. We also calculated target matrix C_3×13_ following the ideal ratio (75% – 13% – 12%) for training-validation-test. The elements of matrix C show the desired number of lesions from different subcategories that must be included within each split. In our search algorithm, we looked for different assignment matrices to reach the minimum weighted average error on the right side (we let the search algorithm to have some level of freedom in number of the patients assigned for training (N *ϵ* (85 ± 2)), validation (N *ϵ* (15 ± 4)), and test (N *ϵ* (14 ± 2))). The subcategories with smaller populations are weighted heavier than the others, to force them to be distributed in a balanced mode. Using a search algorithm to minimize the equation described by Figure 11, we obtained our lesion data splits as described in Figure 3 and Table 1. The equivalent patch distribution demonstrated by Figure 12 indicates favorably balanced number of benign-malignant patches in each split.

## APPENDIX C

### ABLATION STUDY (VALIDATING ResNeXt-50)

In this section, we included the validation results obtained from ResNeXt-50. As can be perceived from Table 11, this model has comparable results with ResNet-50 at all levels with significant improvement utilizing strategy D as discussed in the paper.

## APPENDIX D

### ABLATION STUDY (ResNet-50 VS ResNet-101)

To ensure that we used the right capacity in our design of a classifier, we have compared the performance of ResNet-50 with ResNet-101 which is a widely used tool for lesion classification in literature [31]–[33]. Classification results for both validation and test splits are presented in Tables 12 and 13 where not only we did not see any gains at the expense of a larger architecture, we noticed slightly worse results at a lesion-level especially at the test phase caused by overfitting.

## APPENDIX E

### ABLATION STUDY (WHY 3D ResNet-18?)

In our approach to choose the appropriate design in for 3D analysis of the lesions, we tried 3D ResNet-50 and 3D ResNet-34 as well. Herein, we provided the lesion level results in Table 14 from both validation and test splits using these two methods.

## APPENDIX F

### RESULTS IN FORM OF CONFUSION MATRICES

Herein, we provided the corresponding elements of the confusion matrices for all the experiments in form of Table 13. This table may provide additional information in terms of the performance for each method using various patch or lesion extraction strategies. It should be noted that “Negative” in True Negative or False Negative implies “classified as Benign” and “Positive” in True positive or False positive means “classified as Malignant”.
